# Artificial and natural duplicates in pyrosequencing reads of metagenomic data

**DOI:** 10.1186/1471-2105-11-187

**Published:** 2010-04-13

**Authors:** Beifang Niu, Limin Fu, Shulei Sun, Weizhong Li

**Affiliations:** 1 California Institute for Telecommunications and Information Technology, University of California San Diego, La Jolla, California 92093, USA; 2 Center for Research in Biological Systems, University of California San Diego, La Jolla, California 92093, USA

## Abstract

**Background:**

Artificial duplicates from pyrosequencing reads may lead to incorrect interpretation of the abundance of species and genes in metagenomic studies. Duplicated reads were filtered out in many metagenomic projects. However, since the duplicated reads observed in a pyrosequencing run also include natural (non-artificial) duplicates, simply removing all duplicates may also cause underestimation of abundance associated with natural duplicates.

**Results:**

We implemented a method for identification of exact and nearly identical duplicates from pyrosequencing reads. This method performs an all-against-all sequence comparison and clusters the duplicates into groups using an algorithm modified from our previous sequence clustering method cd-hit. This method can process a typical dataset in ~10 minutes; it also provides a consensus sequence for each group of duplicates. We applied this method to the underlying raw reads of 39 genomic projects and 10 metagenomic projects that utilized pyrosequencing technique. We compared the occurrences of the duplicates identified by our method and the natural duplicates made by independent simulations. We observed that the duplicates, including both artificial and natural duplicates, make up 4-44% of reads. The number of natural duplicates highly correlates with the samples' read density (number of reads divided by genome size). For high-complexity metagenomic samples lacking dominant species, natural duplicates only make up <1% of all duplicates. But for some other samples like transcriptomic samples, majority of the observed duplicates might be natural duplicates.

**Conclusions:**

Our method is available from http://cd-hit.org as a downloadable program and a web server. It is important not only to identify the duplicates from metagenomic datasets but also to distinguish whether they are artificial or natural duplicates. We provide a tool to estimate the number of natural duplicates according to user-defined sample types, so users can decide whether to retain or remove duplicates in their projects.

## Background

Metagenomics is a new field that studies the microbes under different environmental conditions such as ocean, soil, human distal gut, and many others [1-6]. Using culture-independent genomic sequencing technologies, metagenomics provides a more global and less biased view of an entire microbial community than the traditional isolated genomics. The earlier metagenomic studies were largely carried out with Sanger sequencing, but recently, more studies [7-9] were performed with the new breaking through next-generation sequencing technologies [10]. Today, the pyrosequencing by Roche's 454 life science serves as a dominant sequencing platform in metagenomics.

However, it is known that the 454 sequencers produce artificially duplicated reads, which might lead to misleading conclusions. Exact duplicates sometimes were removed before data analyses [7]. Recently, in the study by Gomez-Alvarez et al [11], nearly identical duplicates, the reads that begin at the same position but may vary in length or bear mismatches, were also classified as artifacts. Exact and nearly identical duplicates may make up 11~35% of the raw reads.

In metagenomics, the amount of reads is used as an abundance measure, so artificial duplicates will introduce overestimation of abundance of taxon, gene, and function. Duplicated reads observed in a pyrosequencing run include not only artificial duplicates but also natural duplicates - reads from the same origin that start at the same genomic position by chance. Therefore, simply removing all duplicates might also cause underestimation of abundance associated with naturally duplicated reads. The occurrence of natural duplicates can be very low for metagenomic samples lacking dominant species [11], or can be very high for other samples like transcriptomic samples (results in this study). So it is important not only to identify the duplicates, but also to distinguish whether they are artificial or natural duplicates.

Exactly identical sequences can be easily found, but identification of non-exact duplicates requires sophisticated algorithms to process the massive sequence comparisons between reads. In Gomez-Alvarez et al's study [11], the duplicates were identified by first clustering the reads at 90% sequence identity using cd-hit program [12-14] and then parsing the clustering results.

In this study, we first implemented a method for identification of exact and nearly identical duplicates from pyrosequencing reads. As the original developers of cd-hit, we reengineered cd-hit into a new program that can process the duplicates more effectively than the original program. This method can process a typical 454 dataset in ~10 minutes; it also provides a consensus sequence for each group of duplicates. Secondly, we validated this method using the underlying raw reads from a list of genome projects utilizing pyrosequencing technology. We compared the occurrences of the duplicates identified by our method and the natural duplicates made by independent simulations. Lastly, we studied duplicates for several metagenomic samples and estimated their natural duplicates under different situations.

## Results and discussion

### Program for identification of duplicates

We implemented a computer program called cdhit-454 to identify duplicated reads by reengineering our ultra-fast sequence clustering algorithm cd-hit [12-14]. The algorithm and other details of cdhit-454 are introduced in the Methods section. Briefly, we constrained cdhit-454 to find exact duplicates and nearly identical duplicates that start at the same position and are within the user-defined level of mismatches (insertions, deletions, and substitutions). We allow mismatches in order to tolerate sequencing errors. The default parameters of mismatches are based on the pyrosequencing error model derived in this study. We provide a tool in cdhit-454 to build a consensus sequence for each group of duplicates. The model used for consensus generation is also described in the Methods section. Cdhit-454 software and web server are available at http://cd-hit.org/.

### Duplicated reads of genomic datasets

We tested and validated cdhit-454 with data from pyrosequencing-based genome projects where both the complete genomes and the underlying raw reads are available from NCBI at RefSeq and Short Read Archive (SRA). For a project with multiple sequencing runs, only the run with the most reads was selected. We identified 39 such genome projects on September 2009, with 15 from GS-20 and 24 from GS-FLX platform (including 1 GS-FLX Titanium). The details of the 39 projects, including their project identifiers, SRA accession numbers, genome sizes, GC contents, and other calculated results, are summarized in Table 1.

**Table 1:** Genome projects with full genomes from Refseq and pyrosequencing reads from Short Read Archive

ID^a^	SRA Study^a^	SRA Run^a^	Platform	Genome	Genome size (Mbp)	GC (%)	Number reads	Read Density^b^	% of total Duplicates	% of natural Duplicates (σ ^d^)	
20067	SRP000091	SRR000351	GS_20	NC_010741	1.13946	52	529181	0.4644	13.585	5.032	(0.030)

20739	SRP000868	SRR017616	GS_FLX	NC_013170	1.61780	50	513712	0.3175	17.751	4.999	(0.022)

29525	SRP000571	SRR013433	GS_FLX	NC_013124	2.15816	68	570098	0.2641	9.938	4.464	(0.034)

19265	SRP000036	SRR000223	GS_20	NC_010085	1.64526	34	429372	0.2609	12.120	4.418	(0.026)

19981	SRP000204	SRR001584	GS_20	NC_010830	1.88436	35	399515	0.2120	26.027	4.293	(0.030)

20655	SRP000207	SRR001568	GS_20	NC_012803	2.50109	72	528437	0.2112	9.734	4.099	(0.032)

20833	SRP000867	SRR017612	GS_FLX^e^	NC_013174	2.74965	58	574027	0.2087	16.266	3.745	(0.027)

18819	SRP000035	SRR000219	GS_20	NC_009637	1.77269	33	332809	0.1877	15.145	3.664	(0.024)

29443	SRP000895	SRR017790	GS_FLX	NC_013166	2.85207	43	529344	0.1855	16.017	3.044	(0.025)

29419	SRP000560	SRR013388	GS_FLX	NC_012785	2.30212	41	416146	0.1807	9.537	3.025	(0.031)

19543	SRP000205	SRR001565	GS_20	NC_010483	1.87769	46	321938	0.1714	25.373	2.886	(0.038)

29381	SRP000558	SRR013382	GS_FLX	NC_011832	2.92292	55	461295	0.1578	8.526	2.911	(0.024)

29403	SRP000584	SRR013477	GS_FLX	NC_013162	2.61292	39	400460	0.1532	11.796	2.549	(0.021)

29177	SRP000442	SRR007446	GS_FLX	NC_011901	3.46455	65	438386	0.1265	14.140	2.239	(0.023)

29493	SRP000569	SRR013431	GS_FLX	NC_011883	2.87344	58	362855	0.1262	11.171	2.204	(0.023)

29175	SRP000928	SRR018125	GS_FLX	NC_011661	1.85556	33	225795	0.1216	6.209	2.151	(0.021)

27731	SRP000397	SRR006411	GS_FLX	NC_011769'	4.04030	67	488823	0.1209	18.793	2.073	(0.020)

31289	SRP000919	SRR018042	GS_FLX	NC_012917	4.86291	51	517593	0.1064	3.211	1.948	(0.037)

20635	SRP000049	SRR000266	GS_20	NC_011666	4.30543	63	401125	0.0931	10.364	1.943	(0.022)

31295	SRP000921	SRR018051	GS_FLX	NC_012912	4.81385	54	441287	0.0916	6.938	1.941	(0.019)

29527	SRP000893	SRR017783	GS_FLX	NC_013173	3.94266	58	352814	0.0894	8.356	1.926	(0.022)

20039	SRP000209	SRR001574	GS_FLX^f^	NC_010524	4.90940	68	422674	0.0860	8.566	1.785	(0.023)

19701	SRP000046	SRR000255	GS_20	NC_010644	1.64356	39	136514	0.0830	5.922	1.744	(0.022)

19743	SRP000045	SRR000254	GS_20	NC_011145	5.06163	74	409136	0.0808	7.464	1.739	(0.019)

20095	SRP000054	SRR000278	GS_20	NC_011891	5.02933	74	404796	0.0804	8.363	1.515	(0.022)

30681	SRP000922	SRR018054	GS_FLX	NC_012947	4.57094	50	367491	0.0803	11.324	1.449	(0.018)

21119	SRP000208	SRR001573	GS_FLX^f^	NC_012032	5.26895	56	392222	0.0744	10.026	1.321	(0.025)

18637	SRP000034	SRR000215	GS_20	NC_010172	5.47115	68	395973	0.0723	12.998	1.306	(0.016)

20167	SRP000053	SRR000277	GS_20	NC_011004	5.74404	64	413261	0.0719	14.572	1.248	(0.022)

19989	SRP000211	SRR001579	GS_20	NC_010571	5.95761	65	378824	0.0635	5.484	1.145	(0.023)

19449	SRP000043	SRR000248	GS_20	NC_011768	6.51707	54	395672	0.0607	16.631	1.108	(0.018)

33873	SRP000554	SRR013372	GS_FLX	NC_012691	3.47129	49	191873	0.0552	43.680	1.001	(0.025)

27951	SRP000587	SRR013487	GS_FLX	NC_013132	9.12735	45	496792	0.0544	15.017	0.283	(0.033)

20827	SRP000582	SRR013470	GS_FLX	NC_012669	4.66918	73	246279	0.0527	4.140	0.295	(0.023)

33069	SRP000920	SRR018045	GS_FLX	NC_012880	4.67945	55	226208	0.0483	13.585	5.032	(0.030)

19705	SRP000576	SRR013446	GS_FLX	NC_013093	8.24814	73	381851	0.0462	17.751	4.999	(0.022)

29975	SRP000443	SRR013137	GS_FLX	NC_011992	3.79657	66	161655	0.0425	9.938	4.464	(0.034)

17265	SRP000067	SRR000311	GS_20	NC_008369	1.89572	32	28221	0.0148	12.120	4.418	(0.026)

20729	SRP000267	SRR004103	GS_FLX	NC_012918	4.74581	60	22822	0.0048	26.027	4.293	(0.030)

^a^Project IDs, SRA study accessions, and SRA run accessions are from NCBI Short Read Archive at http://www.ncbi.nlm.nih.gov/Traces/sra/sra.cgi.

^b^Read Density is the number of reads divided by the genome length.

^d^σ is the standard deviation, which is based on the results of 100 simulations (see the "Duplicated reads of genomic datasets" section).

^e^The platform provided by SRA is GS_FLX, and the read length (~400 bp) suggests GS_FLX Titanium.

^f^The platform provided by SRA is GS_20, but the read length (~200 bp) suggests GS_FLX.

For each genome project, we applied cdhit-454 on the raw reads to identify the duplicates, which include both artificial and natural duplicates. The number of natural duplicates was empirically estimated by applying cdhit-454 on simulated reads, which are fragments randomly cut from the complete genomes.

**Figure 1:**
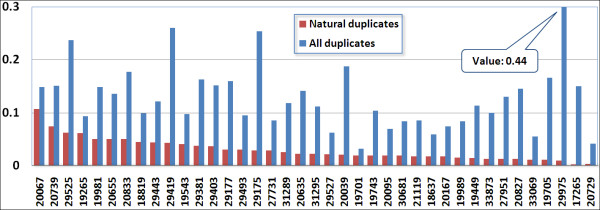
**Ratio of all duplicates and average natural duplicates to all reads from genome projects**. X-axis is project identifier of datasets, which are ordered by decreasing read density (number of reads divided by genome size). Y-axis is the ratio of duplicated reads to all reads.

A simulated reads set and the experimental raw reads set in each genome project have the exactly the same number of sequences of exactly the same lengths. We generated 1000 sets of simulated reads for each genome project and selected 100 sets that are most similar to its corresponding raw reads in GC content. We further introduced sequencing errors (insertions, deletions, and substitutions) to the simulated reads according to the error model derived in this study (Table 2, 3). These processes made the simulated read sets as similar as possible to the real reads set, except that the former only contains natural duplicates. Using cdhit-454, we identified the duplicates for the 100 sets of simulated reads of each project and calculated the average duplicate ratio and the standard deviations. Figure 1 shows the ratio of all duplicates and the average natural duplicates for these 39 projects. The results and the standard deviations are also available in Table 1.

**Table 2:** pyrosequencing error rate of GS-20 platform

		Percentage by error types (%)		
		
Project ID^a^	Error Rate^b^	Insertion	Deletion	Substitution
17265	0.00774	27.06	17.28	55.67
18637	0.00250	60.94	22.24	16.82
18819	0.01194	36.91	19.62	43.47
19265	0.00893	41.32	14.51	44.18
19449	0.00569	38.98	20.23	40.79
19543	0.00522	49.07	18.97	31.96
19701	0.01097	32.89	17.93	49.18
19743	0.00287	57.51	28.29	14.21
19981	0.00530	28.85	17.73	53.42
19989	0.00216	54.29	22.33	23.38
20067	0.00679	46.67	12.15	41.19
20095	0.00216	50.48	28.75	20.77
20167	0.00157	53.65	26.70	19.65
20635	0.00231	57.82	18.25	23.93
20655	0.00211	51.31	29.94	18.75
Average	0.00522	45.85	20.99	33.16

^a^Project ID is the same as in **Table 1**.

^b^Error rate is calculated for aligned reads as the number of errors (insertion, deletion, and substitution) divided by the number of bases of reads.

**Table 3:** pyrosequencing error rate of GS-GLX platform

		Percentage by error types (%)		
		
Project ID^a^	Error Rate^b^	Insertion	Deletion	Substitution
20039	0.00196	51.35	26.49	22.16
21119	0.00360	60.85	22.18	16.97
19705	0.00189	53.24	31.03	15.73
20729	0.00413	19.49	23.86	56.65
20739	0.00244	55.33	24.00	20.66
20827	0.00122	46.32	32.48	21.20
20833	0.00540	53.55	37.38	9.07
27731	0.00280	34.28	19.11	46.61
27951	0.00377	42.11	14.75	43.14
29175	0.00909	40.33	16.96	42.72
29177	0.00645	68.49	16.74	14.76
29381	0.00607	59.57	17.15	23.29
29403	0.01035	58.48	19.48	22.04
29419	0.00689	39.99	17.75	42.26
29443	0.00396	39.97	16.41	43.62
29493	0.00741	46.69	17.81	35.50
29525	0.00196	55.62	28.66	15.72
29527	0.00613	57.44	21.91	20.66
29975	0.00391	57.17	19.47	23.36
30681	0.00605	60.41	20.04	19.55
31289	0.00389	53.18	17.61	29.21
31295	0.00444	58.20	15.56	26.24
33069	0.00508	60.19	18.06	21.76
33873	0.00540	63.31	16.22	20.47
Average	0.00476	51.48	21.30	27.22

^a^Project ID is same as in **Table 1**.

^b^Error rate is calculated for aligned reads as the number of errors (insertion, deletion, and substitution) divided by the number of bases of reads.

As illustrated in Figure 1, the duplicates make up to 4-44% of reads. We observed that the ratio of natural duplicates, which ranges from 1-11%, highly correlates with the read density (number of reads divided by genome size) with a Pearson correlation factor of 0.99. The ratio of artificial duplicates (subtract natural duplicates from all duplicates) varies from 3-42%. On average, artificial duplicates make up 74% of all duplicates, and this percentage varies from 28% to 98% (first left and second right projects in Figure 1). Artificial duplicates happen randomly without observed correlation with the sample's GC content, genome size, or platform (GS-20 or GS-FLX).

Here, we define the sensitivity and specificity for the evaluation of duplicate identification. Within the simulated datasets, the reads that start at the same position are considered as true duplicates. The sensitivity of a method is the ratio of predicted true duplicates by this method to all true duplicates. The specificity is the ratio of predicted true duplicates to all predictions by this method. The averaged sensitivity and specificity for the 39 datasets are both ~98.0% using the default parameters of cdhit-454.

### Pyrosequencing errors

The original 454 publication reported an error rate at ~4% [15]. But later studies yielded much higher accuracy. For example, Huse et al. concluded an error rate at ~0.5% for GS20 system [16]. Quinlan et al. provided a similar error rate at about ~0.4% [17].

Accurate estimation of the pyrosequencing error rate is very important for this study, because we use the error rate to optimize the parameters for cdhit-454 program to identify the duplicated reads with sequencing errors. The error model is also used to guide the generation of sequencing errors in the simulated datasets in above analysis. Therefore, we re-evaluated the pyrosequencing error rate using the data from Table 1.

We used Megablast [18] with the default parameters to align the raw reads back to the corresponding reference genomes. Only the reads with at least 90% of the length aligned were selected to calculate the error rates. Other reads were treated as from contamination material, and therefore discarded. The error rates and fractions by error types (insertion, deletion, and substitution) for all projects are shown in Table 2 for GS-20 and Table 3 for GS-FLX.

We found that the error rate (number of errors divided by total bases) for pyrosequencing is from 0.4% to 0.5%. About 75% of the reads have no error; and about another 20% of the reads have ≤ 2% errors. If the sequencing error rate is 2%, two reads may have up to 4% mismatches. We set the default mismatch cutoff at 4% for cdhit-454 so that about 95% of reads can be covered. We examined several mismatch cutoffs from 95% to 98% on the simulated datasets; the 96% cutoff gave the best sensitivity and specificity. The mismatch cutoff parameter in cdhit-454 is a user-configurable parameter. If the low-quality reads are already filtered out, a higher cutoff such as 98% may be used.

### Duplicated reads of metagenomic datasets

**Figure 2:**
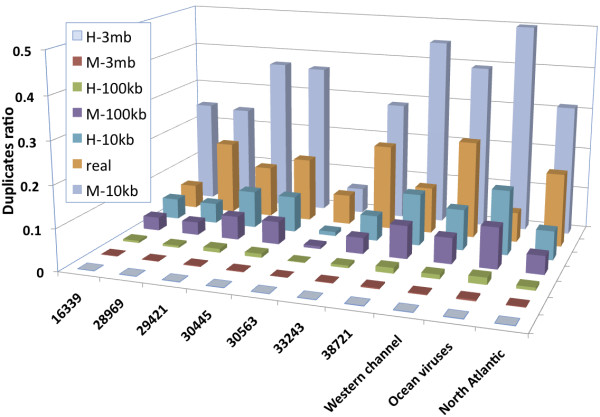
**Ratio of all duplicates and natural duplicates under different hypothetical types for metagenomic samples**. X-axis is the name or project identifier of metagenomic samples. For the real metagenomic dataset, the duplicates include both artificial and natural duplicates. For other hypothetical sample types, the duplicates are natural duplicates.

We studied the pyrosequencing reads for 10 metagenomic datasets (Table 4) of different environments from NCBI SRA or from CAMERA metagenomic project http://camera.calit2.net. We identified their duplicates with cdhit-454 (Figure 2). The duplicates make up 5-23% of reads. As concluded earlier in this study, the quantity of natural duplicates of metagenomic samples depends on the read density of their individual species, and therefore can vary significantly. Since the exact species composition and genome sequences are unknown for metagenomic samples, we could not calculate the amount of natural duplicates as we did for the genome projects. So, we simulated the occurrence of natural duplicates under several hypothetical sample types.

**Table 4:** Metagenomic datasets used in this study

					% of natural duplicates under hypothetical sample types					
					High-complexity^b^			Moderate-complexity^c^		
					
Project/Sample^a^	Environment	Platform	Number Reads	% of total Duplicates	3 mb	100 kb	10 kb	3 mb	100 kb	10 kb
16339/SRR000905	Marine	GS_20	208633	5.74	0.01	0.52	4.98	0.10	3.22	24.88
28969/SRR000674	Coastal water	GS_FLX	201671	17.65	0.02	0.51	4.87	0.10	3.13	24.27
29421/SRR001308	Waste water	GS_FLX	378601	12.39	0.03	0.93	8.94	0.20	5.65	37.09
30445/SRR001663	Marine	GS_FLX	369811	15.39	0.03	0.93	8.68	0.19	5.49	36.53
30563/SRR001669	Human gut	GS_20	41649	7.26	0.00	0.11	1.00	0.03	0.65	6.16
33243/SRR006907	Freshwater	GS_FLX	255722	20.57	0.02	0.61	6.07	0.13	3.88	28.71
38721/SRR023845	Phyllosphere	GS_FLX	543285	11.17	0.05	1.33	12.41	0.29	7.93	45.07
Western channel/Apr_Day_gDNA	Saline water	Titanium	421004	23.38	0.04	1.04	9.80	0.20	6.23	39.42
Ocean viruses/Arctic_Shotgun	Ocean viruses	GS_20	688590	7.14	0.05	1.67	15.46	0.36	9.86	50.15
North Atlantic/BATS-174-2	Ocean gyre	GS_20	288735	17.56	0.02	0.73	6.92	0.16	4.43	31.24

^a^Datasets are either from NCBI Short Read Archive with project IDs and run accession numbers at http://www.ncbi.nlm.nih.gov/Traces/sra/sra.cgi or from CAMERA with project and sample names at http://camera.calit2.net.

^b^High-, ^c^moderate-complexity microbial (or viral) environment with average genome length of 3 mb, 100 kb, and 10 kb

Metagenomic samples are roughly grouped into low-, moderate-, and high-complexity samples, which represent samples dominated by a single near-clonal population, samples with more than one dominant species, and those lacking dominant populations respectively[19]. We constructed 6 hypothetical sample types: M-3 mb, M-100 kb, M-10 kb, H-3 mb, H-100 kb, and H-10 kb; here the name of a sample type starts with M or H (for moderate- or high-complexity) followed by the average genome size. Therefore, M-3 mb represents a moderate-complexity microbial sample with 3 MB genomes; and H-10 kb may represent a high-complexity small viral sample. We assumed that each hypothetical sample contained 100 different genomes of certain length. Given a set of reads, in order to calculate the natural duplicates under a high-complexity hypothetical sample type, we assigned these reads to the 100 genomes randomly at arbitrary positions on either strand. For a moderate-complexity type, 50% of the reads were randomly assigned to 3 dominant genomes, and other reads were randomly mapped to the remaining 97 low-abundance genomes. The natural duplicates were identified by comparing the mapping coordinates. We applied this method to the 10 metagenomic datasets to calculate the ratio of their natural duplicates under different hypothetical sample types (Figure 2 and Table 4).

From Figure 2, we can see that if these metagenomic samples match H-3 mb, M-3 mb, and H-100 kb, their natural duplicates only make up 0.2%, 1.5%, and 7.4% of all duplicates on average; so it is appropriate to filter out the duplicates. However, if a metagenomic sample matches other types, the number of anticipated natural duplicates may be similar or even larger than the artificial duplicates.

Here, we want to discuss a particular type of samples - metatranscriptomic samples. Similar to metagenomics, metatranscriptomics is a field that studies the microbial gene expression via sequencing of the total RNAs extracted directly from environments. Recent metatranscriptomic studies [8,20,21] were performed with pyrosequencing. Since most microbial transcript sequences are only 10^2^~10^4 ^bases in length and one transcript can have many copies in a cell, so the read density of metatranscriptomic samples is several orders of magnitude higher than the read density of metagenomic samples. It is expected that metatranscriptomic samples have high occurrence of natural duplicates. For example, 61% of reads in the mRNA samples from [21] are found as duplicates by cdhit-454; it is reasonable to believe most of them are natural duplicates and therefore should be kept for abundance analysis.

## Conclusions

In this study, we present an effective method to identify exact and nearly identical duplicated sequences from pyrosequencing reads. But since the identified duplicates contain natural duplicates, it is important to estimate the proportion of natural duplicates. In the cdhit-454 package, we provide a tool to estimate the number of natural duplicates under any hypothetical sample type defined by users, so users can decide whether to retain or remove duplicates in their projects.

## Methods

### Algorithm of cdhit-454

In cdhit-454, we use the original clustering algorithm of cd-hit [12-14]. Briefly, reads are first sorted in decreasing length. The longest one becomes the seed of the first cluster. Then, each remaining sequence is compared to the seeds of all existing clusters. If the similarities with existing seeds meet pre-defined criteria, it is grouped into the most similar cluster. Otherwise, a new cluster is defined with the sequence as the seed. The pre-defined criteria includes: (1) they start at the same position; (2) their lengths can be different, but shorter one must be fully aligned with the longer one (the seed); (3) they can only have 4% mismatches (insertion, deletion, and substitution); and (4) only 1 base is allowed per insertion or deletion. Here, (3) and (4) are set according to the pyrosequencing error rate derived in this study (Result and discussion) and can be adjusted by users.

### Generate consensus

We provide a program to build a consensus sequence for each group of duplicates. This tool takes the output of cdhit-454 and original FASTA or FASTQ file. It builds a multiple sequence alignment for each group of duplicates with program ClustalW[22], then generate consensus based on following model:

If a FASTA file is used, the most frequent symbol in each column of the alignment is used as the consensus, and then symbols representing gaps are removed from the consensus sequence. If a FASTQ file is used, the quality score for each base is converted into its error probability to improve the consensus generation.

In a column of a multiple alignment, the count for gaps is calculated as the real count, while the counts for letters {'A','C','G','T'} are "corrected" by the error probabilities, with contribution from letter 'N' which is not counted for itself. Suppose there are *M *letters in one position of a M-sequence alignment: *b(i)*∈ {*'A'*, *'C'*, *'G'*, *'T'*}, *i *= 1, ..., *M *, with quality scores *s*_*i *_and error probabilities *p*_*i*_, where,

The count *c(α) *for α∈ {*'A'*, *'C'*, *'G'*, *'T'*} is calculated as,

where *δ*(*x*, *y*) is a function that takes value one when *x *equals to y, and take zero otherwise. Namely, if the *b*_*i *_is α, a fraction count of *1-p*_*i *_is added for letter α, and a fraction count of *p*_*i *_is equally distributed on the other letters among {'*A'*, *'C'*, *'G'*, *'T'*}, which reflects the nature of the error probability.

Then for each column, if there is a dominant letter or gap with frequency equal to or greater than 0.5, this dominant symbol is used in that column in the consensus, otherwise letter 'N' is used, and then symbols representing gaps are removed from the consensus sequence.

### Estimate natural duplicates in hypothetical metagenomic samples

We provide a program to estimate the number of natural duplicates under any hypothetical sample type. A user provides the number of reads and the size and abundance of genomes in a hypothetical sample. Our tool gives the number of simulated natural duplicates.

## Authors' contributions

BN designed and carried out the study, analyzed the results and wrote the manuscript. LF implemented the method and wrote the manuscript. SS prepared the datasets and analyzed the results. WL conceived of the study, implemented the method and wrote the manuscript. All authors read and approved the final manuscript.
